# Risk factors and outcomes for failure of biological reconstruction after resection of primary malignant bone tumors in the extremities

**DOI:** 10.1038/s41598-021-00092-1

**Published:** 2021-10-14

**Authors:** Taweechok Wisanuyotin, Permsak Paholpak, Winai Sirichativapee, Weerachai Kosuwon

**Affiliations:** Department of Orthopaedics, Faculty of Medicine, Khon Kaen University, Khon Kaen, 40002 Thailand

**Keywords:** Medical research, Oncology, Risk factors

## Abstract

Biological reconstruction is widely used to reconstruct bone defects after resection of bone tumors in the extremities. This study aimed to identify risk factors for failure and to compare outcomes of the allograft, nonvascularized autograft, and recycled frozen autograft reconstruction after resection of primary malignant bone tumors in the extremities. A retrospective study was performed at a single center between January 1994 and December 2017. Ninety patients with primary malignant bone tumors of the extremities were treated with tumor resection and reconstruction using one of three bone graft methods: nonvascularized autograft (n = 27), allograft (n = 34), and recycled frozen autograft (n = 29). The median time for follow-up was 59.2 months (range 24–240.6 months). Overall failure of biological reconstruction occurred in 53 of 90 patients (58.9%). The allograft group had the highest complication rates (n = 21, 61.8%), followed by the recycled frozen autograft (n = 17, 58.6%) and nonvascularized autograft (n = 15, 55. 6%) groups. There was no statistically significant difference among these three groups (*p* = 0.89). The mean MSTS score was 22.6 ± 3.4 in the nonvascularized autograft group, 23.4 ± 2.6 in the allograft group, and 24.1 ± 3.3 in the recycled frozen autograft group. There was no significant difference among the groups (*p* = 0.24). After bivariate and multivariable analyses, patient age, sex, tumor location, graft length, methods, and type of reconstruction had no effects on the failure of biological reconstruction. Biological reconstruction using allograft, nonvascularized autograft, and recycled frozen autograft provide favorable functional outcomes despite high complication rates. This comparative study found no significant difference in functional outcomes or complication rates among the different types of reconstruction.

## Introduction

Choices of bone reconstruction after resection of bone tumors include biological, endoprosthesis, and a combination of biological and endoprosthesis. Endoprosthesis reconstruction provides immediate stability with no disease transmission, but its longevity and cost are the main concerns^1^.

Nowadays, biological reconstruction for the treatment of a large bone defect is increasingly used. The advantages of biological reconstruction include its durability when host-bone graft incorporation is achieved and its cost-effectiveness. The disadvantages include the lengthy time needed to achieve bony union and various complications that require secondary procedures. Choices for biological reconstruction include allograft, vascularized and nonvascularized autograft, bone transportation, and recycled autograft^2^.

Allograft has been used for more than one hundred years. Its limitations are donor availability, size-matching, and disease transmission. Nonvascularized and vascularized autograft can be used in specific locations, such as in small bone defects of the distal radius or large bone defects such as in the resection arthrodesis procedure of the knee or intercalary reconstruction of long bones. Recycled autograft has been increasingly used as an alternative procedure when allograft is not available. The advantages of this method are anatomic conformation, non-transmission of disease, and low cost. Various methods of recycled autograft include autoclaving, irradiation, pasteurization, and freezing with liquid nitrogen^2,3^.

Several previous studies have reported the long-term oncologic and functional outcomes in patients who underwent biological reconstruction with allograft, nonvascularized autograft, and recycled frozen autograft; however, there has been no study focusing on a comparison among these procedures. We thus compared the long-term results and complications of the different methods of bone graft after resection of primary malignant bone tumors of the extremities. We also identified the risk factors that influence the failure of limb salvage after biological reconstruction.

## Patient and methods

Study protocol was approved from our institutional review board (HE631439, Khon Kaen University, Thailand) and informed consent was obtained from all subjects or, if subjects are under 18, from a parent and/or legal guardian. All the protocol was performed in accordance with the relevant guidelines and regulations. We retrospectively reviewed the data of patients who underwent biological reconstruction of the extremities at our tertiary referral center between January 1994 and December 2017. Of the 188 patients whose tumors were managed with biological reconstruction after resection of bone tumors, 31 underwent recycled frozen autograft with liquid nitrogen, 100 with fresh autograft, and 57 with allograft reconstruction. The inclusion criteria were patients treated with biological reconstruction after resection of primary malignant bone tumors of the extremities. The exclusion criteria were patients with inadequate data or followed up for less than 2 years. We excluded 98 patients; 74 had a diagnosis of giant cell tumor of bone, 10 were followed up for less than two years, 8 died from tumor-related causes before the two-year follow-up, and 6 were lost to follow-up. Therefore, 90 patients were enrolled in the study. The median follow-up time was 59.2 months (range 24–240.6 months).

We collected patient age, sex, tumor location, diagnosis, graft length, local recurrence, and mode of graft failure according to Henderson’s classification^4^: Type 1 Soft-tissue failure (1A: failure of function, 1B: failure of cover); Type 2 Graft-host nonunion (2A: hypertrophic, 2B: atrophic); Type 3 Structural failure (3A: fixation, 3B: graft); Type 4 Infection (4A: early, 4B: late); Type 5 Tumor progression (5A: soft tissue, 5B: bone); and, Type 6 Pediatric failure (6A: physeal arrest, 6B: joint dysplasia).

### Surgical procedures

At our institute, biological reconstruction is preferred over endoprosthesis reconstruction due to the economic constraints of the patients. Nonvascularized autograft, allograft, and recycled frozen autograft with liquid nitrogen are the preferred methods for biological reconstruction. The choice of biological reconstruction was made after thorough discussion among the respective surgeon, patient, and patient family. All patients that had been diagnosed with osteosarcoma and Ewing’s sarcoma received chemotherapy according to each protocol before and after surgery (neoadjuvant chemotherapy). Prophylactic antibiotics were given before and after surgery to all patients.

### Nonvascularized autograft

**Figure 1:**
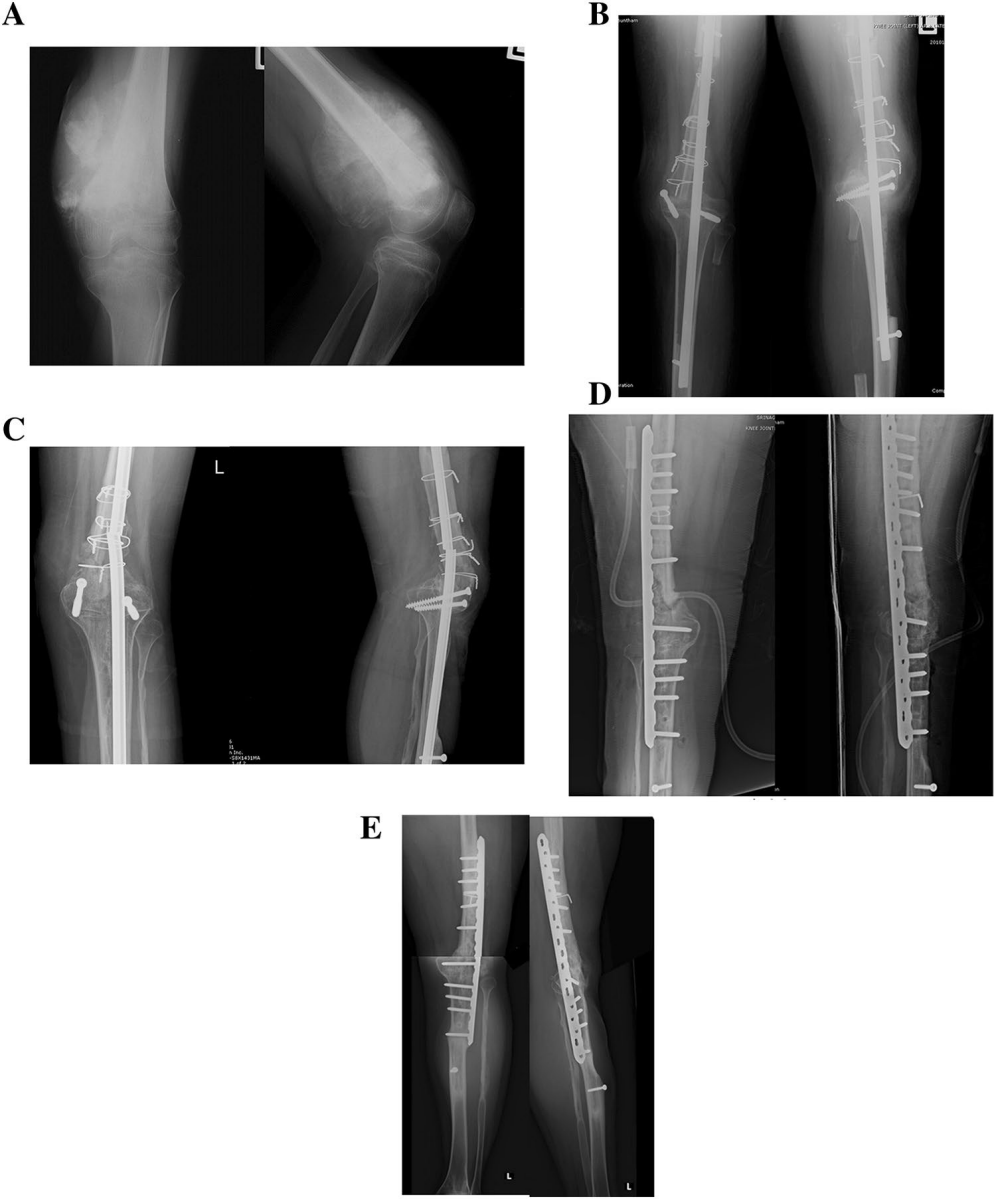
Thirteen-year-old male with osteosarcoma of the distal femur with extensive muscle involvement. (**A**) preoperative anteroposterior and lateral radiograph. (**B**) Postoperative radiograph after wide resection and reconstruction with tibial turn-up and augmentation with nonvascularized fibula graft (resection arthrodesis). (**C**) Break of intramedullary nail occurred 2 years postoperatively. (**D**) Revision surgery with plate and screws. (**E**) Eight years after the index procedure with bone union.

We used nonvascularized autograft reconstruction after resection of bone tumor in the following conditions. (1) For tumors of the distal radius, the ipsilateral or contralateral proximal fibula was used to replace the resected bone. (2) For tumors of the distal femur or proximal tibia, after resection of the tumor, the ipsilateral proximal tibia or distal femur was used, respectively, and augmentation was achieved with an ipsilateral nonvascularized fibula graft to fill the defect as in the intercalary reconstruction of the knee (resection arthrodesis procedure) (Fig. 1). (3) As for intercalary reconstruction of a long bone, the fibula was harvested from the same patient, and the defect was replaced after excision of the tumor.

### Allograft

**Figure 2:**
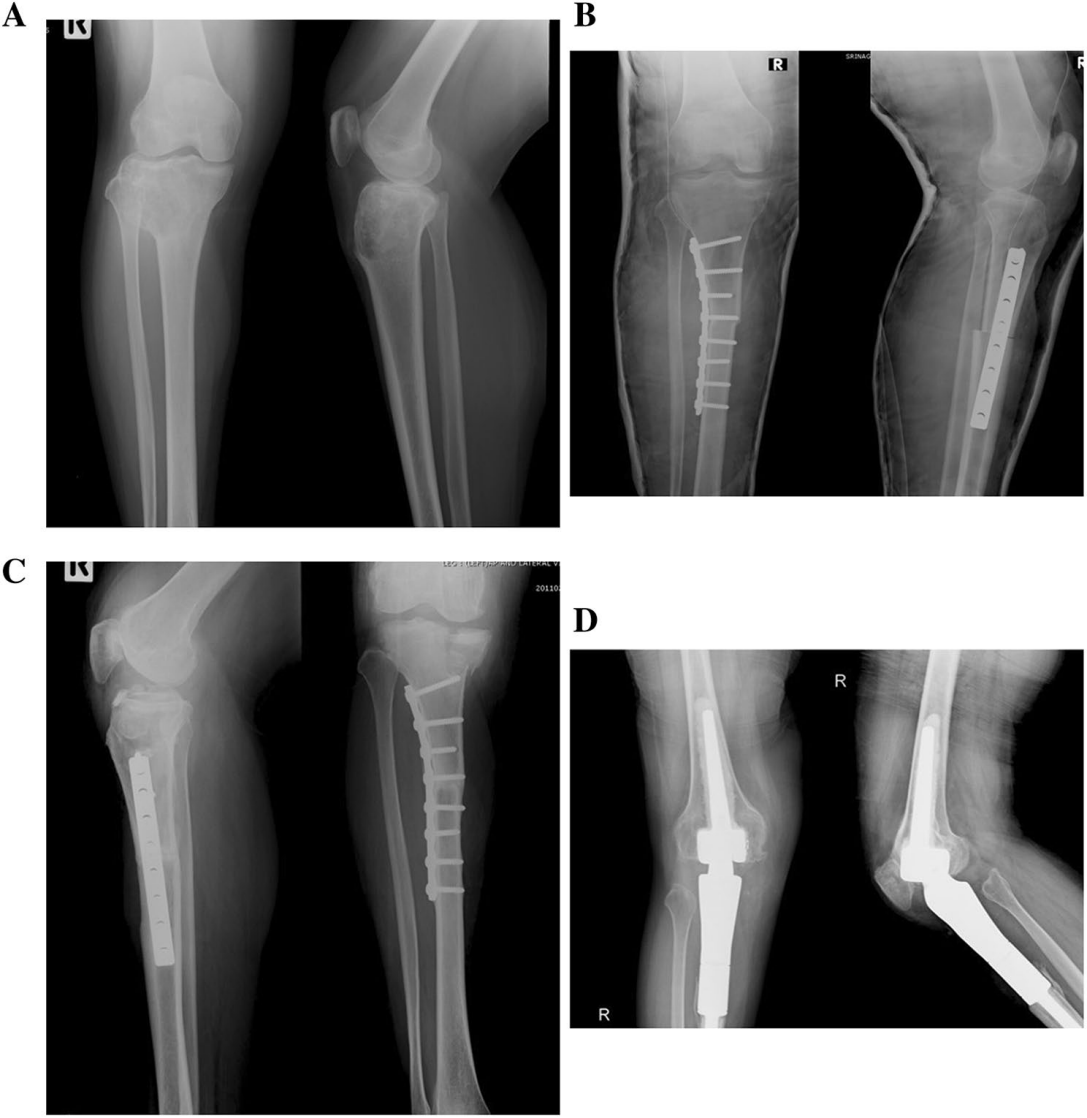
A 33-year-old female with osteosarcoma of the right proximal tibia. (**A**) Pre-operative anteroposterior and lateral radiograph. (**B**) After adjuvant chemotherapy, wide resection and reconstruction with proximal tibia allograft were performed. (**C**) Fracture of allograft (medial tibial condyle) occurred 5 years after reconstruction. (**D**) Revision surgery with proximal tibia endoprosthesis successfully performed.

Allograft had been indicated for bone tumors with osteolytic lesions, and so must be available at the time of surgery. As such, all allografts were harvested under sterile conditions and stored frozen in the bone bank at our institution. Each allograft was thawed in normal saline with antibiotics before fixation to the host bone with plates and screws (Fig. 2). The tendons, ligaments, and joint capsules of the allograft were sutured with nonabsorbable sutures to the respective insertions on the host bone. The extremities were immobilized in casts or braces for at least two months postoperatively or until bone unions were seen from radiographs.

### Frozen autograft with liquid nitrogen

**Figure 3:**
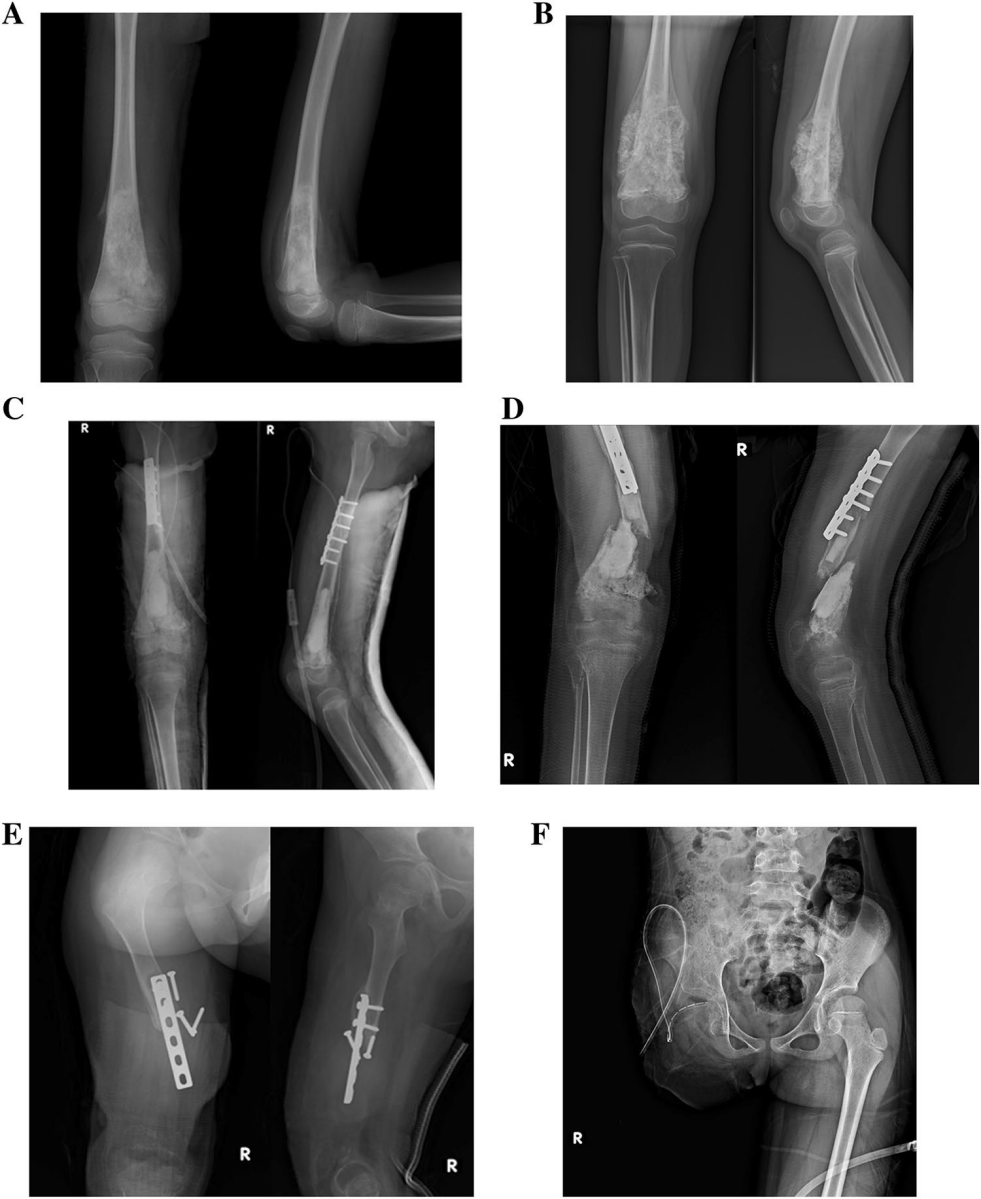
A 6-year-old girl with conventional osteosarcoma of the right distal femur. (**A**) Preoperative anteroposterior and lateral radiograph. (**B**) After chemotherapy, an osteoblastic lesion of the distal femur was observed. (**C**) Wide resection and reconstruction with recycled frozen autograft were performed. (**D**) Fracture of the graft occurred 1 year after surgery. (**E**) Lysis of the graft was found 15 months later. (**F**) Finally, hip disarticulation was performed.

The surgical procedure of frozen autograft with liquid nitrogen was modified from Tsuchiya et al.^4^. We used this method in cases of osteoblastic tumors or when no allograft was available at the time of surgery (Fig. 3). The tendons, ligaments, and joint capsules of the frozen autograft were sutured to the host bone as in the allograft procedure. Postoperative immobilization of the extremity with a brace or cast for at least two months or until the union of the host bone and graft was observed.

### Clinical and radiographic follow-up

Patients were followed up monthly for four months post-operatively, then at 2-month intervals until one year, then every 3 months for two years, and every 6 months after that. Radiographs were evaluated at each follow-up until bone union was achieved. The clinical and functional outcomes were evaluated until the last follow-up using the Musculoskeletal Tumor Society (MSTS) scoring system^5^.

### Statistical analysis

The statistical analysis of the continuous and categorical variables among the three groups was tested using one-way analysis of variance (ANOVA) and the Chi-square test, respectively. If the overall one-way ANOVA was statistically significant, then a post-hoc analysis using Tukey’s test was performed.

The survivorship of the graft was calculated from the date of operation until the date of the first event of the failure of the graft. The overall survival of the graft was calculated using the Kaplan–Meier survival analysis, and a log-rank test was used to compare the difference in survival among the groups.

The Cox proportional hazards regression model was used to determine the independent prognostic factors for failure of biological reconstruction. Independent variables including sex, age, location, length of resection, method, and type of grafts, were analyzed. Crude and adjusted hazard ratios (HRs) with 95% confidence intervals (CIs) were computed in bivariate and multivariable analyses to evaluate the effect of risk factors. Risk factors were considered statistically significant if *p* < 0.05.

All statistical analyses were performed using SPSS version 23 (SPSS Inc., Chicago, IL, USA.).

## Results

Ninety patients with primary malignant bone tumors of the extremities were treated with tumor resection and reconstruction with three methods of bone graft; twenty-seven (30%) underwent reconstruction with nonvascularized autograft, 34 (37.8%) underwent reconstruction with allograft, and 29 (32. 2%) underwent reconstruction with recycled frozen autograft. At the last follow-up, 41.1% (37 of 90) of all patients had died of disease, 25.9% (7 of 27) in the nonvascularized autograft group, 50% (17 of 34) in the allograft group, and 44.8% (13 of 29) in the recycled frozen autograft group. The overall median time of follow-up was 59.2 months (range 24–240.6 months). The demographic data of the patients are presented in Table 1.

**Table 1:** Patient characteristics.

Variable	Nonvascularized autograft (N = 27)	Allograft (N = 34)	Frozen autograft (N = 29)	*P*-value
**Sex**				
Male	17 (63%)	15 (44.1%)	20 (69%)	0.11
Female	10 (37%)	19 (55.9%)	9 (31%)	
**Age (years)**				
Median (range)	23 (7.9–61)	17.26 (12–53.1)	14 (6–45)	0.005
< 20	11 (40.7%)	19 (55.9%)	24 (82.8%)	
≥ 20	16 (59.3%)	15 (44.1%)	5 (17.2%)	
**Follow-up (months)**				
Median (range)	78.2 (24.3–240.6)	54.03 (24–219.6)	37.7 (24.6–179.6)	0.58
**Diagnosis**				
Osteosarcoma	22 (81.5%)	32 (94.1%)	24 (82.8%)	
Chondrosarcoma	3 (11.1%)	0	0	
Malignant GCT	1 (3.7%)	1 (2.9%)	1 (3.4%)	
Adamantinoma	0	1 (2.9%)	2 (6.9%)	
Ewing’s sarcoma	1 (3.7%)	0	2 (6.9%)	
**Location**				
Upper extremity	4	3	1	0.33
Lower extremity	33	31	28	
Proximal humerus	0	2 (5.9%)	1 (3.4%)	
Humeral shaft	2 (7.4%)	0	0	
Distal radius	2 (7.4%)	1 (2.9%)	0	
Proximal femur	0	0	4 (13.8%)	
Femoral shaft	0	1 (2.9%)	1 (3.4%)	
Distal femur	18 (66.7%)	16 (47.1%)	11 (37.9%)	
Proximal tibia	5 (18.5%)	14 (41.2%)	12 (41.4%)	
**Resection length (cm)**				
Mean	14.4 ± 0.7	16.2 ± 0.8	17.7 ± 0.9	0.52
< 15	12 (44.4%)	17 (50%)	7 (24.1%)	
≥ 15	15 (55.6%)	17 (50%)	22 (75.9%)	

### Nonvascularized autograft reconstruction

The mean length of nonvascularized autograft was 14.4 ± 3.5 (range 8.4-20 cm), while the mean union time was 10.5 ± 3.7 months (Tables 1 and 2).

**Table 2:** Oncologic and functional outcomes.

Variable	Nonvascularized autograft (N = 27)	Allograft (N = 34)	Frozen autograft (N = 29)	*p*-value
**Time to union**				
(mean ± SD, months)	10.5 ± 3.7	10.4 ± 3.2	8.6 ± 3.5	0.08
Total failure of reconstruction (n)	15 (55.6%)	21 (61.8%)	17 (58.6%)	0.89
**Type 1 Soft-tissue failure**				
1A (failure of function)	0	3 (8.8%)	3 (10.3%)	
1B (failure of cover)	0	0	0	
**Type 2 Graft-host nonunion**				
2A (hypertrophic)	0	1 (2.9%)	0	
2B (atrophic)	4 (14.8%)	0	0	
**Type 3 Structural failure**				
3A (fixation)	4 (14.8%)	1 (2.9%)	2 (6.9%)	
3B (graft)	1 (3.7%)	7 (20.6%)	6 (20.7%)	
**Type 4 Infection**				
4A (early)	0	0	2 (6.9%)	
4B (late)	1 (3.7%)	3 (8.8%)	1 (3.4%)	
**Type 5 Tumor progression**				
5A (soft tissue)	2 (7.4%)	5 (14.7%)	1 (3.4%)	
5B (bone)	2 (7.4%)	1 (2.9%)	3 (10.3%)	
**Type 6 Pediatric failure**				
6A (physeal arrest)	0	0	0	
6B (joint dysplasia)	0	0	0	
MSTS score (Mean ± SD)	22.6 ± 3.4	23.4 ± 2.6	24.1 ± 3.3	0.24
Graft removal (n)	3	4	8	0.16
Amputation (n)	3 (11.1%)	2 (5.9%)	6 (20.7%)	
Conversion to endoprosthesis	0	2 (5.9%)	2 (6.9%)	

**Figure 4:**
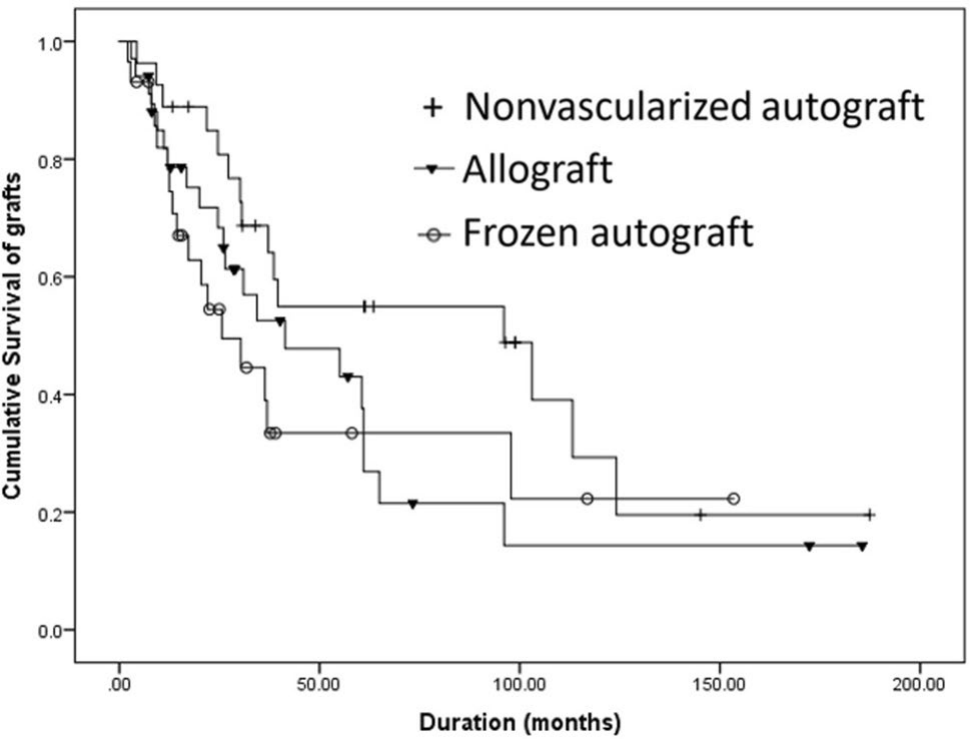
Log-rank survival curve for the three methods of bone graft reconstruction. There was no statistically significant difference between the nonvascularized autograft and recycled frozen autograft groups (*p* = 0.08), the nonvascularized autograft and allograft groups (*p* = 0.12), and the allograft and recycled frozen autograft groups (*p* = 0.63).

The overall 5- and 10-year survival rates of the nonvascularized autograft reconstruction were 54% and 20%, respectively (Fig. 4). Reconstruction failure occurred in 15 of 27 cases (55.6%). The median time from the date of surgery to the date of failure was 30.6 months (range, 4.3-124.1 months). The most common complication was structural failure in five (18.5%), nonunion in four (14.8%), tumor recurrence in four (14.8%), and infection in one (3.7%). Structural failure was mostly due to failure of fixation (four patients), with only one being failure of the graft. All of the patients with structural failure were successfully managed with revision of the implant. In patients with nonunion, an autogenous bone graft from the iliac crest was performed, with the successful bone union being achieved in all cases. Deep infection was managed by aggressive debridement and prolonged intravenous antibiotics. Amputation was needed for three patients after the recurrence of the tumor.

### Allograft reconstruction

The mean allograft length was 16.2 ± 0.8 cm (range 8–31.3 cm), and the mean union time was 10.4 ± 3.2 months (Tables 1 and 2).

The overall 5- and 10-year survival rates of the allograft reconstruction were 22% and 14%, respectively (Fig. 4). The failure of reconstruction occurred in 21 of 34 (61.8%). The median time from the date of surgery to the date of failure was 26 months (range 3–96.1 months).

Structural failure was the most common complication (eight patients). Failure of fixation occurred in one patient (2.9%) and fracture of the allograft in seven (20.6%), followed by tumor recurrence in six (17.6%), infection in three (8.8%), instability in three (8.8%), and nonunion in one (2.9%). Six of the eight cases of allograft fractures were managed by revising the implants, while the other two had to be converted to an endoprosthesis reconstruction. Amputation was needed in two patients due to tumor recurrence.

### Recycled frozen autograft reconstruction

The mean length of recycled frozen autograft was 17.7 ± 0.9 cm (range 8–29 cm), while the mean union time was 8.6 ± 3.5 months (Tables 1 and 2).

The overall 5- and 10-year survival rates of the recycled frozen autograft reconstruction were 32% and 21%, respectively (Fig. 4). Failure of reconstruction occurred in 17 patients (58.6%). The median time from the date of surgery to the date of failure was 14.3 months (range 2.1–124.1 months). Structural failure was the most common complication (eight patients; 27.6%). Of these, two had a failure of fixation and six had a fracture of the allograft. Six patients (20.7%) underwent amputation (two from tumor recurrence, two from persistent deep infection, and one from instability). Two patients had to be converted to an endoprosthesis reconstruction from the graft fracture.

## Comparison among groups of nonvascularized autograft, allograft, and recycled frozen autograft

Time to union in the recycled frozen autograft group (8.6 ± 3.5 months) was faster than in the others groups (10.4 ± 3.2 months in the allograft group and 10.5 ± 3.7 months in the nonvascularized autograft group). There was no statistically significant difference among groups (*p* = 0.08).

The survival analysis on graft failure using the Log-rank test of the three methods of bone graft reconstruction revealed no statistically significant difference between the nonvascularized autograft group and the recycled frozen autograft group (*p* = 0.08), the nonvascularized autograft group and the allograft group (*p* = 0.12), and the allograft group and the recycled frozen autograft group (*p* = 0.63) (Fig. 4).

The allograft and recycled frozen autograft groups had a greater failure rate than the nonvascularized autograft group. The difference, however, was not statistically significant (*p* = 0.89) (Table 2).

Functional outcomes were assessed using MSTS scores, the mean score for nonvascularized autograft, allograft, and recycled frozen autograft group was 22.6 ± 3.4, 23.4 ± 2.6 and 24.1 ± 3.3, respectively. There was no statistically significant difference among groups (*p* = 0.24) (Table 2). However, when a subgroup analysis of non-mobile joint (arthrodesis) and mobile joint (hemiarthroplasty) was performed, the mean MSTS score for arthrodesis and hemiarthroplasty was 21.6 ± 2.5 and 23.8 ± 3, respectively (*p* = 0.01).

At the last follow-up, the grafts were still in place for 88.9% for a median time of 78.2 months in the nonvascularized autograft group, 88.2% for a median time of 54 months in the allograft group, and 72.4% for a median time of 37.7 months in the recycled frozen autograft group. There was no statistically significant difference among groups (*p* = 0.16) concerning the rate of graft removal (Table 2).

Age (*p* = 0.96), sex (*p* = 0.28), tumor location (*p* = 0.3), graft length (*p* = 0.38), method (*p* = 0.32), and type of reconstruction (*p* = 0.24) were not found to have any significant effect on the failure of the biological reconstruction (Table 3).

**Table 3:** Prognostic factors for failure of biological reconstruction.

Variable	Crude			Adjusted*		
	*p*-value	HR	95% CI	*p*-value	HR	95% CI
**Age**						
< 20		1	(reference)		(reference)	
≥ 20	0.67	0.89	0.51–1.54	0.96	0.99	0.54–1.79
**Sex**						
Male		1	(reference)		(reference)	
Female	0.24	1.39	0.8–2.4	0.28	0.72	0.39–1.31
**Tumor location**						
Upper extremity		1	(reference)		(reference)	
Lower extremity	0.35	0.57	0.18–1.85	0.3	2.08	0.52–8.39
**Graft length**						
< 15 cm		1	(reference)		(reference)	
≥ 15 cm	0.99	0.99	0.57–1.73	0.38	0.75	0.4–1.42
**Method of reconstruction**						
Fresh autograft	0.16	1	(reference)	0.32	(reference)	
Allograft	0.14	1.65	0.85–3.22	0.94	0.96	0.32–2.88
Frozen autograft	0.06	1.94	0.96–3.9	0.31	1.59	0.65–3.9
**Type of reconstruction**						
Osteoarticular		1	(reference)		(reference)	
Intercalary	0.08	1.66	0.95–2.9	0.24	0.58	0.24–1.44

HR, Hazard ratio; CI, confidence interval.

*Adjusted for age, sex, tumor location, graft length, method and type of reconstruction.

## Discussion

Large bone defects after resection of bone tumors remain a challenging problem for the surgeon. Biological reconstruction is a widely used procedure to solve this problem. Understanding the risks and benefits of the procedure is necessary for optimum outcomes. To the best of our knowledge, this is the first study to directly compare the long-term outcomes among nonvascularized autograft, allograft, and recycled frozen autograft for reconstruction after tumor resection in patients with primary bone tumors of the extremities.

In the current study, the median age of patients in the frozen autograft group was younger than the others groups. The skeletally matured patients trended to receive a nonvascularized autograft and allograft treatment because of readily size-matched bone and allograft in the bone bank. By contrast, among skeletally-immature patients, size-matching of allografts was more difficult so frozen autografts were more commonly selected for this patient group.

Nonvascularized autograft is considered the reference standard as it provides histocompatibility, osteogenicity, osteoconductivity, and osteoinductivity^6^. Allografts have variable osteoinductive and osteoconductive properties but have lower osteogenic potential than autografts^7^. Takata et al. studied the effect of temperature on osteoinductivity and found that bone morphologic activity (osteoinductive properties) was better preserved in frozen autografts than in those treated with autoclaving or pasteurization. Igarashi et al.^3^ also reported that frozen autografts had both osteoinductive and osteoconductive properties.

The overall survival of the allograft, as reported by Muscolo et al., was 78% after 5 and 10 years^8^. Wu et al.^9^ reported that the 5- year survival rate of frozen autograft was 83%. Igarashi et al.^3^ reported that the respective overall 5- and 10-year survival rate of recycled frozen autograft reconstruction was 86.1% and 80.6%. Schuh et al. reported that the 5- and 10-year survival rate for nonvascularized autograft was 47%^10^.

In the current study, the survival rates of grafts were worse than those reported in other studies. In our series, the respective 5- and 10-year survival rates for all three groups ranged from 22 to 54% and 14 to 21%. The explanation for the lower rate might be due to the difference in the definition of “failure of biological reconstruction”. We used the definition proposed by Henderson et al.^11^ while other studies defined failure as graft removal or needed a revision procedure or an amputation^3,12–14^.

The mean length of nonvascularized bone graft in our series was 14.4 ± 0.7 cm and the mean union time was 10.5 ± 3.7 months. Our data confirmed that nonvascularized bone graft could be used for reconstruction of bone defects longer than 6 cm^15^. Liu et al.^16^ reported 26 patients with giant cell tumors of the distal radius treated with resection and reconstruction with nonvascularized fibula autograft. In that series, the mean length of the fibular graft was 8.3 cm and union was achieved in an average of 8.4 months. There was no case of fracture or failure of internal fixation. Krieg et al.^17^ evaluated 31 patients treated with nonvascularized fibular grafts after resection of primary musculoskeletal tumors. They reported a high rate of graft union (89%) in a median union time of 6 months. Fracture of the graft occurred in 19% of their cases.

Enneking et al.^18^ reported the results of resection arthrodesis in patients with benign aggressive and primary malignant bone tumors around the knee. The complications of massive autogenous bone graft used in distal femur or proximal tibia were nonunion in 4 of 20 patients (20%) and structural failure in 5 of 20 patients (3 fracture of the graft, 1 failure of the implant, and 1 fracture of the graft and implant). Campanacci et al.^19^ found that infection was the only major complication that occurred in 5 of 26 patients (19.2%), and nonunion occurred in 2 patients (7.7%). In our series, comparable to previous studies, we found a high rate of union (85.2%) among patients who underwent reconstruction with nonvascularized autogenous bone graft with a structural failure of 18.5% and an infection rate of 3.7%.

Many authors have reported a high rate of allograft failure. Mankin et al.^20^ reported 718 allograft procedures in patients with benign and malignant bone tumors. Fracture was the most common complication (19%), followed by nonunion (17%), infection (11%), and unstable joint (6%). Fracture and infection usually occurred within 3 years and 1 year after surgery, respectively. Brigman et al.^14^ reported failure rates between 11 and 56% for osteoarticular and intercalary types of femur and tibia. Donati et al.^21^ retrospectively reviewed 112 patients with high grade osteosarcoma: the allografts used included arthrodesis (n = 44), intercalary graft (n = 39), osteoarticular graft (n = 22), and allograft prosthesis composite (n = 7). High complication rates included delayed union (49%) and fracture (27%). Bus et al.^13^ studied 87 cases of intercalary allograft reconstruction. Complications occurred in 76% of patients including nonunion (40%), fracture (29%), and infection (14%). Ogilvie et al.^22^ reviewed 20 patients who underwent reconstruction with osteoarticular allograft after extremity sarcoma resection. All patients were followed up for at least 10 years. They reported a high rate of complications (70%), fracture in nine cases, progressive arthritis in five, nonunion in four, and infection in two. The rate of allograft removal was 60%. The present study showed a high complication rate (61.8%) among patients who had allograft reconstruction. Major complications included structural failure (23.5%) followed by tumor recurrence (17.6%), instability (8.8%), and infection (8.8%). Our data are comparable to previous studies; despite a high complication rate, a favorable functional outcome for the patients in this group was demonstrated.

Igarashi et al.^3^ reported a series of 36 patients who underwent reconstruction with recycled frozen autograft and a complication rate of 39%, including a fracture rate of 19.4%, a tumor recurrence rate of 11.1% and an infection rate of 11.1%. By comparison, Wu et al.^9^ reported a failure rate as high as 40% (34 from 85 patients), with nonunion (13%) being the most common complication followed by tumor progression (11%), structural failure (8%), infection (5%), and soft tissue failure (4%). Hindiskere et al.^23^ reported on 41 patients using recycled frozen autograft for treatment of malignant bone tumors, 16 (39%) had postoperative complications, and six (15%) underwent revision surgery. Complications included six infections, four graft resorptions, two nonunions, and one delayed union. Abdel Rahman et al.^24^ studied 10 patients with osteosarcoma who underwent reconstruction with recycled frozen autograft; skin sloughing and delayed union occurred in one case; and a high mean functional score was 82.4% was reported. In the present study, the results were similar to previous studies. The overall complication rate for recycled frozen autograft was 58.6%, with the most common complications being structural failure (27.6%), tumor recurrence (13.7%), infection (10.3%), and instability (10.3%). Our mean functional score was 24.1 (80.33%). Several methods can be used to reduce the structural failure after frozen autograft reconstruction: (a) pedicle-freezing technique^25^; (b) double-plate fixation or intramedullary nail with plate fixation to increase stability of the osteotomy sites; and, (c) bone cement augmentation to increase the strength of the processed bone^2^. The local recurrence rate in our series was 13.7% and is comparable to previous studies which reported rates between 2.4 and 16.7%^3,9,23,25–29^. The risks for local recurrence using the frozen autograft technique include bone tumor with substantial soft tissue extension, tumor close to the neurovascular sheath^3^, pedicle freezing technique^23^, and chemotherapy resistant patients^30^. Regarding tumor recurrence, especially in the case of soft tissue extension that may result in local recurrence from adjacent soft tissue. Tsuchiya et al.^30^ recommended taking precautions when using the recycled technique, such as using surgical sheets to protect adjacent tissues, performing curettage carefully, and using operative instruments separately to reduce the possibility of tumor contamination.

Several authors have studied the risk factors for failure of biological reconstruction. Schuh et al.^10^ assessed 27 patients with vascularized and 26 patients with non-vascularized autologous fibular grafts for reconstruction of a diaphyseal bone defect after resection of a musculoskeletal tumor. The risk factors for revision were vascularized fibula graft reconstruction, a short length of the graft, and a fibular graft in the lower limb. Adjuvant chemotherapy, age, and fixation method (plate, external fixator, or K-wire) did not have a statistically significant influence. Brigman et al.^14^ evaluated types of femoral and tibial allografts (allograft-arthrodesis, allograft-prosthesis, intercalary, and osteoarticular) and found no significant difference in graft survival between groups. In contrast, Aponte-Tinao et al.^12^ found that osteoarticular tibial grafts had a higher risk of graft failure than those of femoral allografts.

Hazan et al.^31^ studied the effect of chemotherapy on osteoarticular allografts. Patients who received chemotherapy had a greater rate of nonunion than the non-chemotherapy group (32% versus 12%). The effect of chemotherapy on nonunion was confirmed by Hornicek et al.^32^ who found that the nonunion rate increased to 27% versus 11% of patients who did not receive chemotherapy. Donati et al.^21^ reported that chemotherapy increased the risk of delayed union but that it had no effect on infection or fracture.

Muscolo et al.^8^ analyzed 62 distal femoral osteoarticular allografts of benign aggressive and malignant bone tumors. Age, sex, percentage of the resected femur, and chemotherapy did not significantly affect the overall allograft survival rate.

The variables identified in the present study were age, sex, tumor location, graft length, method, and type of reconstruction. These variables did not have a significant effect on the failure of biological reconstruction. We did not include chemotherapy as one of the variables for risk of graft failure because most of the patients received chemotherapy, so a calculation of the role of chemotherapy was not possible.

We found no significant difference in complication rates and functional outcomes among the three methods of reconstruction. Choosing an appropriate method of biological reconstruction for each patient depends on several factors: (1) type of bone lesion—for osteoblastic lesions, recycled bone is an appropriate choice, while for extensive osteolytic lesions, the allograft or autograft is preferred over frozen autograft; (2) the availability of bone bank and donors are prerequisites for allograft preparation; and, (3) surgeon expertise and preference are essential for successful outcomes.

The study has several limitations. First, this was a retrospective study, so the range of diagnoses, different methods of fixation, and various regimens of chemotherapy could have influenced the outcomes. Second, there was a small number in each group due to the rarity of the disease and procedures. A larger sample size study should be conducted to confirm the effect of these procedures. Third, surgical techniques and types of implants have improved over time, which may reduce the failure rate.

## Conclusions

Reconstruction after resection of the malignant bone tumors with nonvascularized autograft, allograft, and recycled frozen autograft yielded no difference in functional outcomes or complications. These procedures provide favorable functional outcomes, but a high risk of complication rates should be considered.

## Data Availability

The data that support the findings of this study are available on reasonable request from the corresponding author.
